# A Systematic Review of the Adverse Effects of Long-Term Proton Pump Inhibitor Use on the Gastrointestinal System in the Adult Population

**DOI:** 10.7759/cureus.90606

**Published:** 2025-08-20

**Authors:** Sandeep Sekar Lakshmisai, Roshitha S Bheemaneni, Evangeline C Nwachukwu, Aahana Nigam, Priyanka Sakarkar, Safeera Khan

**Affiliations:** 1 Department of Medicine, SRM Prime Hospital, Chennai, IND; 2 Gastroenterology, AdventHealth Ocala, Ocala, USA; 3 Internal Medicine, Gandhi Medical College, Secunderabad, IND; 4 Health Emergency Preparedness and Response, Nigeria Centre for Disease Control and Prevention, Abuja, NGA; 5 Cardiology, Trinity College Dublin, Dublin, IRL; 6 General Surgery, Princess Royal University Hospital, Orpington, GBR; 7 Family Medicine, The Michener Institute of Education at UHN (University Health Network), Toronto, CAN

**Keywords:** adult population, adverse effects, gastrointestinal system, long-term use, proton pump inhibitors

## Abstract

Proton pump inhibitors (PPIs) have been the first-line drug of choice for acid-peptic diseases for a long time. While literature is abundant on the efficacy of the drug, the adverse effects of this drug, especially in the gastrointestinal (GI) system, have been a topic of ongoing research interest. The long-term safety profile remains controversial. PPIs have been found to cause polyps, malignancies, and pre-cancerous conditions such as intestinal metaplasia, enteric infections such as *Clostridium difficile*, and microscopic colitis. Furthermore, long-term use is seen to be associated with decreased iron absorption and disruption in gut microbiota. This systematic review aims to perform a qualitative synthesis of the literature on the long-term adverse effects of PPIs on the GI system. This review utilized the Preferred Reporting Items for Systematic Review and Meta-Analyses (PRISMA) 2020 guidelines. PubMed, PubMed Central (PMC), MEDLINE (Medical Literature Analysis and Retrieval System Online), MDPI (Multidisciplinary Digital Publishing Institute), and EBSCO (Elton B. Stephens Company) Library databases were searched for data published in the past 15 years, between 2010 and 2024, using Medical Subject Headings (MeSH) and keywords, and a total of 260 articles were identified. The PubMed search, which included articles from MEDLINE and PMC, yielded 216 results; the MeSH search included 11 articles. EBSCO and MDPI searches retrieved nine and 24 articles, respectively. After applying the eligibility criteria and performing qualitative synthesis, 50 articles were shortlisted. Quality assessment tools, such as the Assessment of Multiple Systematic Review (AMSTAR) for systematic reviews, the Cochrane bias assessment tool for randomized control trials, the scale for the assessment of narrative review articles (SANRA) checklist for narrative reviews, and the Newcastle-Ottawa Scale (NOS) for observational studies, and JBI critical appraisal checklist for case reports, were used. This review will help clinicians evaluate the risk-benefit factors of PPI use on a case-by-case basis, help mitigate unnecessary long-term prescription of PPI, and reduce the incidence of unwanted adverse events.

## Introduction and background

Proton pump inhibitors (PPIs) are the first-line medications for acid peptic diseases such as gastroesophageal reflux disease (GERD), peptic ulcers, duodenal ulcers, and Zollinger-Ellison syndrome. They are the first-line drugs for the treatment of functional dyspepsia [1]. This drug is also used in quadruple therapy for *Helicobacter pylori*, along with bismuth, amoxicillin, and tetracycline in people with antibiotic resistance [2]. Long-term PPIs are prescribed for conditions such as reflux esophagitis [3]. The association of PPI and mortality and morbidity has been well studied in the United Kingdom using a prospective analysis, demonstrating that PPI is unrelated to all-cause and cause-specific mortality [4].

While PPIs are usually safe when administered for a short time, long-term usage has been an area of concern [5]. A study by Mafi et al. showed that one in eight persons was categorized in the low-value PPI prescription category, and eliminating such incidences can reduce detrimental adverse effects, especially in adults [6]. PPIs have caused duodenal dysbiosis, and long-term supplementation reduced eosinophil levels in the duodenum. [7].

While the benefits of PPIs are well known, the harmful effects have been an area of ongoing investigation. It is crucial to determine whether there are any detrimental effects on the body, especially in the long run. A multicenter study conducted by Kurlander et al. has shown that a causal link exists to many adverse effects, such as enteric infection, polyps, and hypomagnesemia [8]. Usage of PPIs has caused small intestinal bacterial overgrowth [9]. The alteration in gut microbiota has enhanced immunological reactivity and autoimmunity, thus paving the way for several diseases like celiac disease and gut inflammation [10]. Chronic usage of PPIs has been linked with many serious pathologies, such as gastric lymphoma and adenocarcinoma. It was found that the masking of *H. Pylori *infection and long-term infection had caused gastric mucosal atrophy [11].

A retrospective study conducted by Snir et al. indicated that there was a dose-related increase in gastric metaplasia incidence [12]. This was linked to the upper quartiles of cumulative PPI doses (Q4 and Q3 vs. Q1): adjusted odds ratios (OR) 1.32 (95%CI 1.11-1.57) and 1.27 (95%CI 1.07-1.52) for the entire cohort (P_total_ 0.007, P_trend_ 0.013); 1.69 (95%CI 1.23-2.33) and 1.40 (95%CI 1.04-1.89) for *H. pylori*-positive patients (P_total_ 0.004, P_trend_ 0.005); and 1.21 (95%CI 0.98-1.49) and 1.20 (95%CI 0.96-1.49) for *H. pylori*-negative patients (P_total_ 0.288, P_trend_ 0.018).

In this systematic review, we aimed to identify the notable adverse effects of long-term usage of PPIs such as pantoprazole, omeprazole, esomeprazole, rabeprazole, and lansoprazole on the gastrointestinal (GI) system in the adult population globally by analyzing multiple studies.

## Review

Methods

Search Sources and Search Strategy

We used the Preferred Reporting Items for Systematic Review and Meta-Analyses (PRISMA) 2020 guidelines to conduct the research [13]. Our search included databases such as PubMed, including MEDLINE (Medical Literature Analysis and Retrieval System Online) and PubMed Central (PMC), EBSCO (Elton B. Stephens Company), and databases hosting MDPI (Multidisciplinary Digital Publishing Institute) journals. We searched for studies published between 2010 and 2024. Keywords such as proton pump inhibitors, adverse effects, gastrointestinal system, and long-term use were used in combinations in the search. However, a PubMed-specific search strategy was developed using Medical Subject Headings (MeSH): ("Proton Pump Inhibitors"[MeSH] OR "Omeprazole"[MeSH] OR "Esomeprazole"[MeSH] OR "Pantoprazole"[MeSH]) AND ("Adverse Effects"[Subheading] OR "Drug-Related Side Effects and Adverse Reactions"[MeSH]) AND ("Time Factors"[MeSH] OR "Chronic Disease"[MeSH]) AND ("Gastrointestinal System"[MeSH] OR "Gastric Mucosa"[MeSH] OR "Esophagus"[MeSH] OR "Small Intestine"[MeSH] OR "Colon"[MeSH]) AND ("Adult"[MeSH] OR "Middle Aged"[MeSH] OR "Aged"[MeSH]).

Table 1 displays the articles found in each database after the search.

**Table 1:** Search stratergy MeSH: Medical Subject Headings; MDPI: Multidisciplinary Digital Publishing Institute; EBSCO: Elton B. Stephens Company

Search Strategy	Database	Number of articles identified
("Proton Pump Inhibitors"[MeSH] OR "Omeprazole"[MeSH] OR "Esomeprazole"[MeSH] OR "Pantoprazole"[MeSH]) AND ("Adverse Effects"[Subheading] OR "Drug-Related Side Effects and Adverse Reactions"[MeSH]) AND ("Time Factors"[MeSH] OR "Chronic Disease"[MeSH]) AND ("Gastrointestinal System"[MeSH] OR "Gastric Mucosa"[MeSH] OR "Esophagus"[MeSH] OR "Small Intestine"[MeSH] OR "Colon"[MeSH]) AND ("Adult"[MeSH] OR "Middle Aged"[MeSH] OR "Aged"[MeSH])	PubMed (MeSH)	11
Proton pump inhibitor AND adverse effects AND Gastrointestinal system	MDPI	24
Proton pump inhibitor AND adverse effects AND long-term use	EBSCO	9
Proton pump inhibitor AND adverse effects AND Gastrointestinal system	PubMed	216

Eligibility Criteria

Inclusion criteria: Full-text articles in English published in the last 15 years, studies with only human participants, that is, adults over 18 years, on long-term use of PPI were included. For this review, long-term PPI use is defined as continuous or regular use of PPIs for at least eight weeks. However, most included studies evaluated durations of six months to several years, and adverse effects were predominantly reported with use beyond six months.

Exclusion criteria: Studies that included the pediatric population, articles whose full text was not retrieved, grey literature, and papers unrelated to PPI's long-term adverse effects on the GI system were excluded.

Process of Selection

We collected the articles using the keyword search strategy, transferred them to EndNote (Clarivate Plc, London, United Kingdom), and identified the duplicates. Each article with titles and abstracts was screened, and eligibility criteria were applied; articles were then assessed for full-text articles, and selected accordingly. Two reviewers independently screened articles (ECN and AN), and discrepancies were resolved by discussion.

Quality Check of Articles

All the shortlisted articles were screened for quality checks using the relevant quality assessment tools. Systematic reviews were checked by the assessment of multiple systematic reviews (AMSTAR) tools [14], randomized controlled trials (RCTs) were checked by the Cochrane risk of bias assessment tool (RoB-2) [15], narrative reviews by the Scale for the Quality Assessment of Narrative Review Articles (SANRA) checklist [16], cohort studies by the Newcastle-Ottawa Scale (NOS) [17] and, case reports by the JBI Critical Appraisal Checklist for Case Reports [18]. A minimum score of 60% was required for inclusion, ensuring that only studies with acceptable quality were considered in this systematic review. Two authors independently assessed the quality appraisal (SSL and RSB).

This review was not registered on the International Prospective Register of Systematic Reviews (PROSPERO) due to timing constraints. Due to substantial heterogeneity in the study designs, populations, outcome measures, and data reporting across the included studies, a meta-analysis was not conducted.

Results

**Figure 1:**
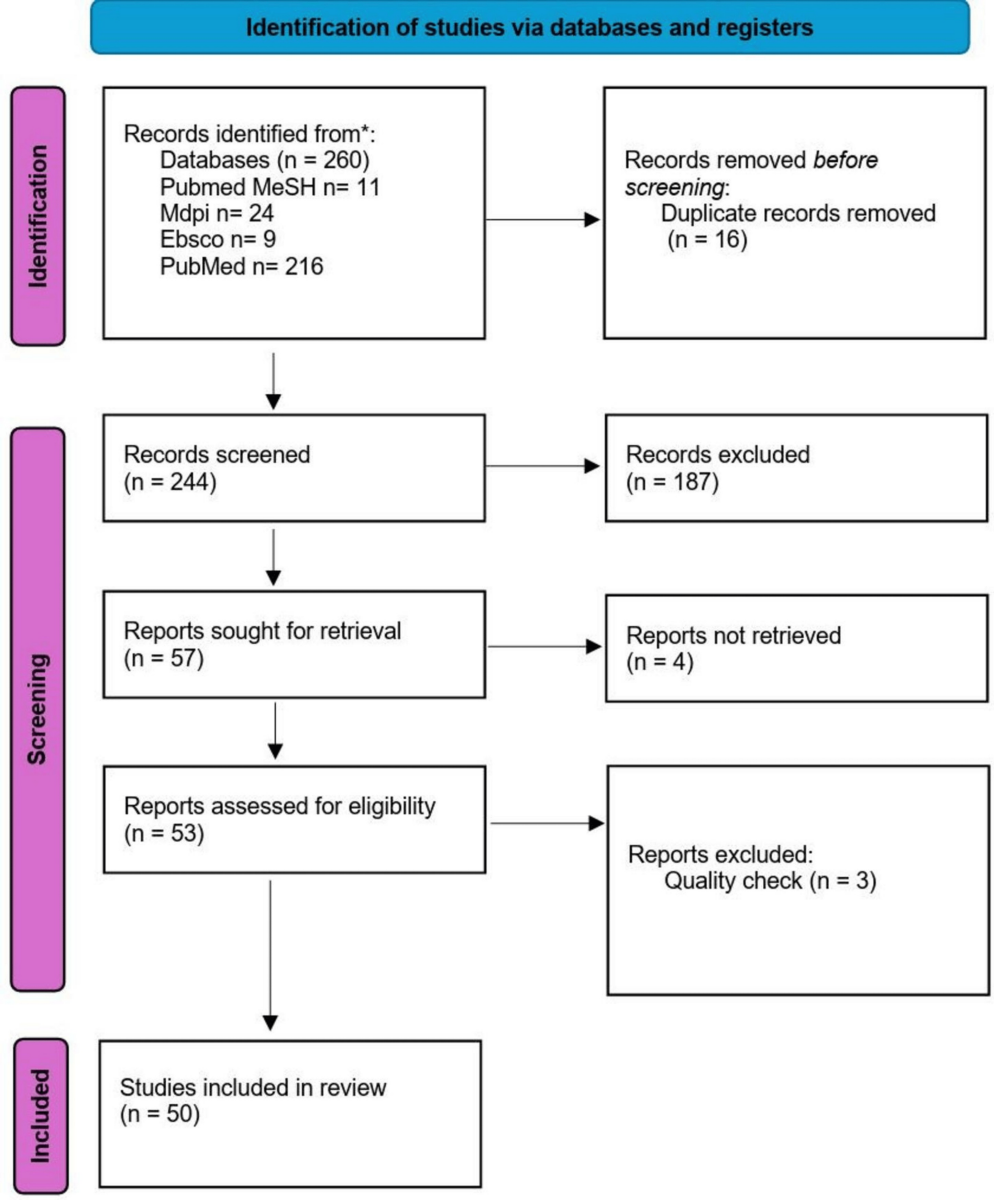
PRISMA flowchart of the selection process PRISMA: Preferred Reporting Items for Systematic Reviews and Meta-Analyses

We identified a total of 260 articles after searching the databases. From these, a total of 16 duplicates were identified through EndNote and removed, and after screening the remaining articles by looking into the titles, abstracts, and full-text availability, 53 articles were shortlisted. These shortlisted full-text articles were assessed for eligibility criteria and underwent quality checks using the quality assessment tools, and finally, 50 articles were finalized for the review [1-12,19-56]. Figure 1 displays the PRISMA flow chart presenting the entire process of identifying, screening, and including all relevant articles.

Outcomes Measures

Data were systematically reviewed from various studies, like RCTs, non-RCTs (NRCTs), observational studies, such as cohort and case reports, and narrative reviews, to determine the association between long-term usage of PPIs on the GI system in the adult population. Primary outcomes included adverse effects on the GI system, such as alteration in the gut flora, lymphoma causation, gastric adenocarcinoma, and iron absorption, which were reviewed and assessed. Secondary outcomes included dysbiosis and mortality. This study aims to provide insights into how long-term administration of this drug could be associated with several detrimental changes in the GI system.

Study Characteristics

The 50 studies reviewed included 31 cohort studies, four RCTs, four systematic reviews and meta-analyses, 10 reports, and one narrative review (Table 2). The sample sizes of the included studies were collected, accounting for the number of participants in each category, such as the participants who used PPIs, the control groups, or the placebo groups. The duration of the studies with PPI users was also collected. The specific PPIs used, including dosage, frequency, and duration of the treatment, were collected. Control groups and placebo groups were assessed. The statistical methods used for data analysis, encompassing descriptive and inferential statistics and measures of effect size, were reported to facilitate result interpretation and determine the significance of the findings.

**Table 2:** Included studies exploring the long-term adverse effects of PPIs on the GI system in adults COVID-19: corona virus disease-19; OR: odds ratio; CI; confidence interval, QIIME: quantitative insights into microbial etiology; NSAID: nonsteroidal anti-inflammatory drug; COX-2: cyclooxygenase-2; CgA: chromogranin A; VCE: video capsule endoscopy: HR: hazard ratio; CDDD: cumulative defined daily dose; aHR: adjusted hazard ratio; GNL: gastric neoplastic lesion; FU: follow-up; AAG: autoimmune atrophic gastritis; CDI*: Clostridioides difficile* infection; SIFO: small intestinal fungal overgrowth; SIBO: small intestinal bacterial overgrowth; HLA: human leukocyte antigen; ECL: enterochromaffin-like cell; AE: adverse events; UGIB: upper gastrointestinal bleed; DDD: defined daily dose; FD:  functional dyspepsia; IQR: interquartile range; LR-BAC: low dose of rabeprazole, bismuth, amoxicillin, and clarithromycin; LR-BAT: low dose of rabeprazole, bismuth, amoxicillin, and tetracycline; HR-BAC: high dose of rabeprazole, bismuth, amoxicillin, and clarithromycin; HR-BAT: high dose of rabeprazole, bismuth, amoxicillin, and tetracycline; GERD: gastroesophageal reflux disease; RE: reflux esophagitis; MDP: minimal distending pressure; MCV: mean corpuscular volume; MWFL: multiple white and flat elevated lesions; PCP' primary care physicians; GCLL: gastric cobblestone lesion; GPA: gastro-protective agents; PP: per protocol; ITT: intention to treat; KCNQ1: potassium voltage-gated channel subfamily Q member 1; aRR: adjusted relative risk; IDA: iron deficiency anemia; TIBC: total iron binding capacity; PPI: proton pump inhibitor; GI: gastrointestinal

Author, year	Type of Study	Purpose of Study	Number of Participants	Results	Conclusions
Al Ali et al., 2022 [34]	Cohort study	Effect of long-term supplementation of omeprazole on hematological and biochemistry profiles.	90	The results showed that patients who took omeprazole had a significant decline in serum ferritin (p<0.0001), vitamin D3 (p<0.01), and calcium levels (p<0.001) than the healthy group.	Long-term omeprazole use is linked to anemia and a decrease in red blood cell indices. It also affects absorption and results in low vitamin and mineral levels.
Al-Momani and Aolymat, 2024 [35]	Cohort study	Association of PPI and gastrointestinal symptoms in patients diagnosed with COVID-19.	254	In comparison to non-PPI users, patients on PPIs had a lower risk of developing myalgia (OR 0.5, 95% CI: 0.3 to 0.9, p = 0.02) but significantly higher odds of developing diarrhea (OR 2.0, 95% CI: 1.08 to 3.93, p = 0.02) and abdominal pain (OR 2.0, 95% CI: 1.22 to 3.93, p = 0.03).	This study showed that patients who took omeprazole during COVID-19 illness had more gastrointestinal effects.
Bajaj et al., 2014 [32]	Randomized Controlled Trial	The linkage between Omeprazole and a shift in gut microbiota in patients diagnosed with cirrhosis.	30	A remarkable microbiota change was seen in controls and those with cirrhosis after omeprazole (QIIME P < 0.0001). *Streptococcaceae* abundance, normally found in increased quantity in saliva, significantly increased after omeprazole in controls (1 vs. 5%) and cirrhosis (0 vs. 9%) and was correlated with serum gastrin levels (r = 0.4, P = 0.005).	Omeprazole is linked to a shift in the microbiota in individuals with compensated cirrhosis and a functional alteration in the distal gut that may pave the way for bacterial overgrowth.
Boghossian et al., 2017 [30]	Systematic Review	Continuing versus deprescribing PPI in the adult population.	1758	On-demand therapy resulted in a clinically meaningful decrease in "drug burden," as indicated by weekly PPI pill consumption (mean difference (MD) -3.79, 95% CI -4.73 to -2.84), supporting deprescribing based on middling quality evidence (four studies, n = 1152). Additionally, there was less evidence that using PPIs on-demand would result in worse participant satisfaction than using them continuously.	Deprescribing led to an increase in Gastrointestinal adverse effects, such as dyspepsia, in patients diagnosed with mild gastroesophageal reflux disease.
Boyce et al., 2015 [33]	Randomized Controlled Trial	Comparison between Netazepide and PPI in Gastric Acid Suppression and Whether Netazepide Can Prevent the Trophic Effects of Hypergastrinemia by PPI.	30	Pentagastrin-stimulated stomach acid secretion was similarly reduced by all treatments. Serum gastrin rose with all treatments, although rabeprazole and the combination increased it more than netazepide. Additionally, the combination decreased the release of basal acids. While netazepide and the combination decreased plasma CgA, rabeprazole increased it.	Netazepide and rabeprazole were equally effective for gastric acid suppression, and Netazepide was effective for the trophic effects of PPI-induced Hypergastrinemia.
Brunner et al. 2012 [36]	Cohort Study	Safety and Efficacy of long-term treatment with pantoprazole in severe acid-peptic disease.	142	Healing rates were 95.8% at 12 weeks. Mean fasting gastrin levels increased from baseline to moderate values throughout long-term therapy. The mean enterochromaffin-like cell density increased moderately during the first three years before stabilizing. Clinically significant alterations in the stomach mucosa were linked to these alterations.	Daily PPI therapy for 15 years for patients with severe acid-peptic disease was found to be effective, and no concerns were found.
Contaldo et al., 2019 [37]	Observational study	Small intestine lesions in patients with iron deficiency anemia detection by video capsule endoscopy.	109	Of the 109 individuals, 80 (73.4%) had VCE abnormal images, whereas the remaining 29 (26.6%) had normal findings. There were 116 lesions in total among the 80 patients with VCE anomalies. We found that 14.5% of the 80 individuals had more than one lesion.	Video capsule endoscopy provided clear information on the broad spectrum of small intestine lesions in patients with iron deficiency anemia.
De Roza et al., 2019 [38]	Retrospective cohort study	Determining whether PPI increases mortality in patients with cirrhosis.	295	The mortality rate was greater for PPI users than non-users [adjusted HR = 2.10, (1.20-3.67); P = 0.009]. Compared to non-users, longer PPI use with cDDD > 90 was linked to greater mortality [aHR = 2.27, (1.10-5.14); P = 0.038]. Hospitalization for hepatic decompensation was more common among PPI users [aRR = 1.61, (1.30-2.11); P < 0.001].	PPI was found to be associated with increased mortality and hepatic failure in cirrhotic patients.
Dilaghi et al., 2022 [39]	Prospective cohort study	To find the impact of PPI on the development of gastric neoplasms in patients with Autoimmune Atrophic Gastritis.	105	There was a positive correlation between PPI use before AAG diagnosis (OR 9.6, 95%CI 2.3-40.3) and the development of GNLs at FU when using logistic regression. However, there was no correlation between other independent variables such as smoking habit (OR 0.4, 95%CI 0.1-2.1), age ≥ 50 years (OR 2.0, 95%CI 0.2-18.1), first-degree family history of gastric cancer (OR 2.4, 95%CI 0.4-15.2), or the use of antiplatelets/anticoagulants (OR 2.8, 95%CI 0.7-12.0).	PPI use was found to increase gastric neoplasms when used before the diagnosis of the Autoimmune Atrophic Gastritis condition.
Freedberg et al., 2013 [40]	Retrospective cohort	To establish the risk between PPI and recurrent *Clostridium difficile* among inpatients.	894	A total of 23% of the cohort experienced a CDI recurrence. PPI use while receiving CDI treatment did not correlate with a recurrence of *C. difficile* (HR 0.82; 95% CI 0.58–1.16). Recurrence ofCDI was linked to Black race (HR 1.66, 95% CI 1.05–2.63), age (HR 1.02, 95% CI 1.01–1.03), and comorbidities (HR 1.09, 95% CI 1.04–1.14). We also examined the subgroup of patients who lived to 90 days of follow-up because that group had a higher 90-day death rate than those who took PPIs (log-rank p = 0.02). PPIs and CDI recurrence were not related once more (HR 0.87; 95% CI 0.60–1.28).	There was no risk between PPI use and recurrent *Clostridium Difficile* Infection in the inpatients.
Fujimoto and Hongo, 2011 [3]	Nonrandomized clinical trial	To determine the safety and efficacy of long-term maintenance therapy with 10mg Rabeprazole once daily in Japanese patients with reflux esophagitis.	192	During the 104 weeks, the endoscopic non-relapse rate for RE was 7.3%. The mean change from baseline in the GERD symptom score following treatment was negative, indicating an improvement in GERD symptoms. Atrophy was determined to have formed in almost no cases, and treatment was safe.	Long-term maintenance with an oral dose of rabeprazole 10 mg was effective.
Hashimoto et al., 2014 [19]	Case report	Iron deficiency anemia due to PPI	1	Physical examination of the patient showed pallor and spoon nails. Hemoglobin dropped to 8.7 g/dL. MCV dropped to 66 (fL). Iron was 21 μg/dL. TIBC was elevated to 382 μg/Dl. The stool occult blood test was negative. Esophagogastroduodenoscopy showed severe atrophy and intestinal metaplasia, and IDA was reported to improve post-rabeprazole discontinuation.	Anemia from PPI was found to be uncommon.
Hatano et al., 2016 [41]	Cohort study	Association of PPI and black spots on the fundic gland region.	26620	PPIs were taken by 68.8%. The black dots were only found around the fundic glands. A total of 41 patients (64.1%) had more than ten black spots. The two types were black spots on the flat mucosa and black spots on the fundic gland polyps. Pathological examination revealed that 26 (76.5%), 23 (67.6%), and 6 (17.6%) patients had observed parietal cell protrusions, fundic gland cysts, and brownish pigmentation in fundic gland cysts, respectively.	The association between black spots and PPI was found to be true.
He et al., 2021 [4]	Prospective cohort study	Association between PPI and risk of all-cause, cause-specific mortality.	440840	Regular PPI use was not associated with all-cause mortality, mortality from neoplasms, circulatory system diseases, respiratory system diseases, digestive system diseases, external causes, and other causes after controlling for confounders like overall health status and chronic illnesses.	No association between PPI and all-cause and cause-specific mortality risk was found.
Herzig et al., 2011 [42]	Cohort study	Association of acid-suppressive medication and upper Gastrointestinal bleeding nosocomial.	78394	The group exposed to acid-suppressive medication had an adjusted odds ratio for nosocomial GI bleeding of 0.63 (95% CI 0.42-0.93) compared to the unexposed group after propensity score matching. Seven hundred and seventy patients had to be treated to stop one nosocomial GI bleeding incident.	Despite acid-suppressive medication's protective impact, the number needed to treat to avoid one nosocomial GI bleeding was considerable, confirming the guideline against routine usage in noncritically ill hospitalized patients.
Horvath et al., 2019 [43]	Observational study	Survival prediction using biomarkers for oralization during long-term PPI in cirrhosis.	90	Long-term PPI was significantly associated with Streptococcus *salivarius*, *Veillonella parvula*, and the genus *Streptococcus* and performed well as biomarkers for PPI-associated dysbiosis (accuracy: 74%, 72%, and 74%, respectively)	Gut-derived indicators of PPI-associated dysbiosis are connected to worse outcomes and may help assess long-term PPI medication adverse effects.
Iida et al., 2012 [44]	Observational study	Association of PPI and inhibition of perception of gastric distension.	10	Post omeprazole administration, abdominal feeling scores for the same incremental pressures over MDP were 0.3 ± 0.1, 0.8 ± 0.1, 2.0 ± 0.4, 2.8 ± 0.4, 3.8 ± 0.4, 4.6 ± 0.4, 4.9 ± 0.3, 5.4 ± 0.4, 5.2 ± 0.6, and 5.0 ± 1.0, respectively. A significant decrease in feeling score was observed at intrabag pressures of MDP + 2 mmHg (P = 0.028) and + 4 mmHg (P = 0.013) after omeprazole.	Omeprazole was found to decrease mechanosensitivity to gastric distension.
Imai et al., 2018 [20]	Case report	IDA due to long-term usage of PPI.	1	Blood work showed red blood cell (RBC) count, 4.51×1012/L with 0.82 % reticulocytes; Hb, 9.9 g/dL; hematocrit (Ht), 32.5 %; and MCV, 72.1 fL. Serum iron, 21 μg/dL; total iron binding capacity, 518 μg/ dL; and ferritin, 8.7 ng/mL, following the diagnosis of iron deficiency anemia	PPI can cause IDA by suppressing gastric acid secretion, thus leading to decreased dietary iron absorption.
Jacobs et al., 2013 [9]	Observational study	PPI association with an increase in Small intestinal bacterial/fungal overgrowth.	150	63% had overgrowth,40% had shown SIBO, 26% had revealed SIFO, and 34% had shown mixed SIBO/SIFO. SIBO was found to be predominantly due to *Streptococcus*, *Enterococcus*, *Klebsiella*, and *E. coli*. SIFO was caused due to Candida. PPI use (P = 0.0063) was a significant risk factor (P < 0.05) for overgrowth.	Dysmotility and PPI use were identified as independent risk factors for SIBO and SIFO in most patients.
Jang et al., 2020 [10]	Randomized Controlled Trial	To study the changes in the gut microbiome and celiac disease serology in patients who take PPIs.	12	One person developed a remarkable increase in the celiac disease-specific autoantibody response to transglutaminase 2 in conjunction with enhanced immune reactivity to gluten. Genotyping revealed positivity for the celiac disease-associated HLA-DQ2 and HLA-DQ8 alleles.	There is potential enhancement of gluten immunopathology and changes in gut microbiome.
Jianu et al., 2012 [21]	Case report	Association between long-term PPI use and gastric carcinoids	2	A routine upper gastrointestinal endoscopy of the two patients taking PPI for 12–13 years revealed a solitary tumor in the oxyntic mucosa. Histology demonstrated a well-differentiated neuroendocrine tumor. Biopsies showed hyperplasia of ECL cells. The tumor regressed in patient two, and the ECL cell hyperplasia regressed in both patients post-PPI discontinuation.	Hypergastrinemia caused by proton pump inhibitors may induce enterochromaffin-like cell carcinoids.
Konijeti et al., 2013 [22]	Case report	Lansoprazole-related microscopic colitis	1	A colonoscopy showed mild patchy erythema in the proximal colon and a linear ulcer in the descending colon of more than 15 cm. Sloughing of the surface epithelium and an increase in intraepithelial lymphocytes were signs of slightly active colitis in the right and left colonic biopsies.	Lansoprazole caused microscopic colitis.
Kubo et al., 2020 [23]	Case report	Vonoprazan-associated gastric mucosal redness.	4	Esophagogastroduodenoscopy (EGD) demonstrated linear or spotty redness in the greater curvature of the middle gastric body post-vonoprazan initiation, which disappeared after stopping the drug.	Vonoprazan was found to be associated with gastric redness.
Kurlander et al., 2020 [8]	Observational study	Physician's belief in the adverse effects of PPI.	799	Most considered that PPIs increase the risk for 6 of the 12 AEs reported. However, 79% incorrectly suggested stopping PPI use in a high-risk UGIB prevention scenario where long-term use is advised. In the latter case, maintaining PPI was highly linked to perceived effectiveness for preventing bleeding (odds ratio 7.68, P < 0.001 for moderately effective; odds ratio 17.3, P < 0.001 for extremely successful).	Most Internists strongly believed that PPI caused numerous adverse effects.
Kwon et al., 2014 [45]	Retrospective cohort study	Association between gastric acid suppressants and peritonitis in patients undergoing peritoneal dialysis (PD).	398	Proton pump inhibitor use was not linked to PD-related peritonitis; only H2-blockers (H2B) was linked to an elevated risk of PD-related peritonitis.	PPI was not found to cause peritonitis in patients undergoing PD
Lassalle et al., 2022 [46]	Case-control study	PPI linkage to pancreatic cancer.	23321	An increase in the risk of pancreatic cancer was linked to ever (as opposed to never) using PPIs [adjusted OR (aOR) = 1.05, 95% CI, 1.01–1.09]. 181–1,080 cDDD: aOR = 1.18, 95% CI, 1.12–1.24; >1,080 cDDD: aOR = 1.17, 95% CI, 1.10–1.23; 31–180 cDDD: aOR = 1.05, 95% CI, 1.00–1.11; 1–30 cumulative defined daily dose (cDDD): aOR = 0.92, 95% CI, 0.87–0.97.	PPI and its association with pancreatic cancer require further studies.
Lué and Lanas, 2016 [29]	Narrative review	Assessing Risks vs benefits of PPI in lower intestinal bleeding.	-	A multivariate analysis showed that concomitant PPI use caused mucosal injury (OR = 2.04; 95% CI: 1.05-3.97).	PPI demonstrated more small bowel damage in the lower gastrointestinal system.
Mafi et al., 2019 [6]	Observational study	Incidence of low-value PPI prescription in the health system among older adults.	399	Of the 399 prescriptions, 143 (35.8%; 95% CI = 31.3%-40.7%) had potentially low values, with 82% starting correctly (for example, GERD) but having a long-term, non-guideline-based pattern. 32 PCPs (18.9%) out of 169 were responsible for 59.2% of prescriptions that might be low value.	1/3rd of the prescriptions was found to be low value.
Majima et al., 2018 [47]	Retrospective cohort study	Association between PPI and white, flat, elevated lesions in the stomach.	767	The prevalence rate of MWFLs was 10.4%; logistic regression analysis revealed the following risk factors: use of PPIs [odds ratio (OR), 3.51; 95% confidence interval (CI), 1.92-6.43], a 1-year increase in age (OR, 1.05; 95% CI, 1.02-1.08), and female sex (OR, 1.92; 95% CI, 1.19-3.12).	PPIs are a risk factor for flat and raised white lesions in the stomach.
Miyamoto et al., 2017 [26]	Case report	PPI induced Endoscopic findings of the gastric mucosa.	1	Following two years of esomeprazole treatment, an esophagogastroduodenoscopy was conducted. Endoscopic findings showed gastric cobblestone-like mucosa in the gastric body after eliminating *Helicobacter pylori*. Oval crypt opening dilatation was detected by endoscopy. A thick gastric second layer and sporadic small a-echoic areas were found.	Endoscopy confirmed that oxyntic gland dilatations raised the stomach mucosa, forming a cobblestone appearance, due to proton pump inhibitors.
Nagao et al., 2024 [27]	Case report	Association of multiple Gastric Neuroendocrine Tumors with Long-term Use of a Proton Pump Inhibitor.	1	Esophagogastroduodenoscopy found three neuroendocrine tumors (NETs) in the gastric body.	PPI was found to be associated with multiple Gastric Neuroendocrine Tumors.
Nakashima et al., 2024 [28]	Case report	Association of PPI with Multiple Gastric Hyperplastic Polyps.	1	A stomach esophagogastroduodenoscopy revealed blood and black residue. There were several hyperplastic polyps that caused the hemorrhage. Following the end of PPI, EGD revealed that the polyps had nearly vanished.	PPI was found to be associated with multiple gastric hyperplastic polyps
Nasser et al., 2015 [11]	Retrospective cohort study	Effect of PPI on pathological gastric changes.	300	Additionally, multivariate analysis has revealed the identification of *H. pylori* was less likely in prior PPI exposure (OR = 0.217, 95%CI: 0.123-0.385), GERD (OR = 0.317, 95%CI: 0.132-0.763, P = 0.01), and alcohol consumption (OR = 0.396, 95%CI: 0.195-0.804, P = 0.01). A considerable decrease in *H. pylori* densities and an increased risk of intestinal metaplasia result from long-term PPI use, which may conceal *H. pylori* infections and encourage the development of non-*H. pylori* gastritis diagnosis.	PPI use promotes the incidence rates of intestinal metaplasia, hides *H. pylori* infection, and identifies chronic gastritis that is not caused by *H. pylori *infection.
Paroni Sterbini et al., 2016 [48]	Observational study	Effect of PPI on the gastric mucosa microbiota	24	98% of all sequences belonged to* Fusobacteria* and *Actinobacteria*, with *Helicobacter*, *Streptococcus*, and *Prevotella* among the top 10 most prevalent genera. The makeup of stomach microbial species in dyspeptic individuals was not substantially impacted by *H. pylori* infection or PPI therapy. Additionally, a significant rise in *Streptococcus* was observed concerning PPI medication; this increase appeared to occur irrespective of *H. pylori* infection.	Streptococcus is a vital marker of changes in the composition of the gut microbiota brought on by PPIs.
Parsons et al., 2017 [49]	Observational study	Comparing the Human Gastrointestinal Microbiota in Hypochlorhydric states due to Proton Pump Inhibitor use.	1400	PPI-treated patients showed relatively few alterations in the gastric microbiota compared to healthy subjects.	Despite serum gastrin concentrations comparable to *H. pylori*-induced atrophic gastritis patients, PPI therapy did not significantly affect the gastric flora.
Rodrigues et al., 2024 [50]	Cross-sectional study	Assessment of Long-Term PPI among Older Portuguese Adults in Primary Care.	1200	In the older population, 37.92% were using PPIs, and 78.68% were using them for longer than recommended. Also, 49.79% were taking PPIs without having any indication. Multivariate analysis demonstrated that the long-term use of PPIs was not associated with any specific pattern, but inappropriate PPI use was high among Portuguese older adults.	Long-term usage of unnecessary PPI was noted, which needed to be regulated.
Shanika et al., 2023 [5]	Systematic Review	Global Trends and Patterns of PPI.	28 million	Around 25% of adults were using PPI. Sixty-three percent of PPI users were under 65. 75% of PPI users were "White" ethnic, while 56% were female. Of those who took PPIs, nearly two-thirds were on high doses (≥ defined daily dose (DDD)), 25% remained for more than a year, and 28% persisted for more than three years.	PPI use must be regulated carefully to prevent unnecessary usage.
Snir et al., 2021 [12]	Retrospective Cohort Study	Assessing Dose-Dependent Association of PPI with Intestinal Metaplasia in *H. Pylori* Positive Patients.	14147	Gastric intestinal metaplasia was reported in 1244 (8.8%) of the 14,147 individuals included (median age 63.4 years; women 54.4%; *Helicobacter pylori*-positive 29.0%). Gastric intestinal metaplasia diagnosis was linked to the upper quartiles of cumulative proton pump inhibitor doses (PPI-Q4 and PPI-Q3 vs. PPI-Q1): adjusted odds ratios were 1.32 (95% CI 1.111.57) and 1.27 (95% CI 1.07-1.52) for the entire cohort (Ptotal 0.007, Ptrend 0.013), 1.69 (95% CI 1.23-2.33) and 1.40 (95% CI 1.04-1.89) for *Helicobacter pylori*-positive patients (Ptotal 0.004, Ptrend 0.005), and 1.21 (95% CI 0.98-1.49) and 1.20 (95% CI 0.96-1.49) for *Helicobacter pylori*-negative patients (Ptotal 0.288, Ptrend 0.018).	Dose-dependent association of PPI with intestinal metaplasia in *H. pylori*-positive patients exists.
Takahari et al., 2017 [51]	Observational study	Formation of Gastric Cobblestone Lesion in the Stomach due to PPI.	171	Of the 171 patients, 60 (35.1%) had GCLLs, and 111 (64.9%) did not.	Gastric cobblestone lesions occurred in people taking PPI, especially those with atrophic gastritis.
Takeda et al., 2017 [24]	Case report	Hemorraghic polyps due to long-term PPI administration.	1	Polyps displayed stromal edema, fundic gland expansions, and hyperplasia in the foveolar epithelium under a microscope. Additionally, parietal and main cell growth was noted. From the base to the top of the mucosa, immunohistochemical analysis revealed dilated mucous glands and parietal cells that were positive for aquaporin-4 (AQP4) and KCNQ1. The emergence of lesions linked to the prolonged use of PPIs was consistent with these findings.	Long-term PPI was strongly associated with Hemorrhagic polyps.
Tatsuguchi et al., 2020 [52]	Prospective Cohort Study	Determining if Long-Term Proton Pump Inhibitor Medication Causes Hypergastrinemia and ECL Cell Cancer in Human Gastric Mucosa.	20	None of the 20 patients evaluated during the PPI treatment period had any cases of gastric epithelial neoplasia or Neuroendocrine tumor.	The relation between PPI and secondary Hypergastrinemia was unclear.
Tranberg et al., 2021 [53]	Observational Study	Oropharyngeal Microbiota disruption due to PPI.	134	Proton pump inhibitor medication and receiving antibiotics before hospitalization was associated with the development of a disturbed oropharyngeal microbiota with colonization of gut pathogens (OR 3.49 [1.19-10.2] and OR 4.52 [1.13-18.1], respectively), while acute hospital admission was associated with a lower risk of colonization of gut pathogens (OR: 0.23 [0.074-0.72]).	PPI was linked with disruption in oropharyngeal microbiota
Valkhoff et al., 2012 [54]	Case-control study	Assessing the risk of Gastroprotection during cyclooxygenase 2 Inhibitor Treatment and the Risk of Upper Gastrointestinal Tract Events.	14416	A UGI tract incident occurred in seventy-four patients during or soon after a period of coxib medication during which a GPA was co-prescribed; the incidence rate was 11.9 (95% CI 9.4-14.8) per 1,000 years of coxib treatment. Patients with less than 20% adherence to GPAs had a 1.97 (95% CI 0.84-4.60) risk of UGI tract events, while those with more than 80% had a higher risk. UGI tract incidents were 9% more likely for every 10% drop in GPA adherence (OR 1.09 [95% CI 1.00-1.18]).	Decreased gastroprotection during COX 2 inhibitor treatment caused increased upper gastrointestinal tract adverse events.
Wauters et al., 2021 [7]	Prospective Cohort Study	PPI and its Link to Duodenal Dysbiosis in Functional Dyspepsia	58	Despite the discontinuation of long-term PPI therapy in FD-stoppers, Streptococcus remained elevated and was linked to duodenal PPI effects in controls.	Long-term PPI was associated with duodenal dysbiosis.
Wu et al., 2014 [25]	Case report	Gastric acid suppression induced fatal Spontaneous *Clostridium septicum* Gas Gangrene	1	Tissue gas gangrene and myonecrosis were confirmed by post-mortem examination. Also, multiple intestinal ulcers containing* Clostridium septicum* were present at autopsy.	PPI has been linked with pathological infection with *Clostridium septicum* gas gangrene.
Xie et al., 2017 [55]	Observational study	To estimate the Risk of Death due to PPI.	6524434	PPI had a higher death risk compared to H2 blocker across a median of 5.71 years (HR 1.25, CI 1.23 to 1.28). The two-stage inclusion estimation (HR 1.21, CI 1.16 to 1.26), the 1:1 time-dependent propensity score-matched cohort (HR 1.34, CI 1.29 to 1.39), and high-dimensional propensity score (HR 1.16, CI 1.13 to 1.18) all showed an increased risk of death linked to PPI usage. When comparing PPI usage to nil use (HR 1.15, CI 1.14 to 1.15), and PPI use to no PPIs (HR 1.23, CI 1.22 to 1.24), the risk of death increased.	The long-term use of PPIs increased the death risk significantly.
Xie et al., 2018 [2]	Randomized Controlled Trial	Determining Ten-Day Efficacy of Quadruple Therapy as First-Line Treatment for* Helicobacter Pylori* infection in high antibiotic-resistant patients.	431	In the LR-BAC, LR-BAT, HR-BAC, and HR-BAT groups, the corresponding per-protocol (PP) eradication rates were 94.1%, 91.9%, 94.8%, and 91.9%, whereas the corresponding ITT eradication rates were 87.2%, 87.2%, 87.7%, and 86%. The four groups did not differ significantly in the ITT analysis (P = 0.985) or the PP analysis (P = 0.799).	Quadruple therapy was an effective first line for *H. Pylori* infection in patients with high antibiotic resistance.
Yamada et al., 2017 [56]	Retrospective Cohort Study	Determining the Association of PPI and Small Bowel Injury	327	Propensity matching based on 327 participants showed no significant differences in the prevalence of small-bowel injuries, including erosions and ulcers, between users and non-users of PPIs.	PPI therapy did not increase the prevalence of small bowel injury.
Pinto-Sanchez et al., 2017 [1]	Systematic review	Use of PPI in functional dyspepsia	8759	PPI therapy was more potent than placebo, with 30% of the PPI group reporting no symptoms, whereas the placebo group reported 25% (RR of remaining dyspeptic 0.88, 95% CI 0.82 to 0.94; P < 0.001, random‐effects model) with a Number Needed to Treat for the benefit of 13.	PPI was effective for dyspepsia and slightly better than H2 blockers.
Song et al., 2014 [31]	Systematic Review and Meta-Analyses	Association between long-term use of PPI and gastric lesions	1789	Based on the meta-analysis, individuals receiving PPI maintenance treatment had a higher likelihood than controls of developing either diffuse (simple) ECL hyperplasia (OR 5.01; 95% CI 1.54 to 16.26; P value = 0.007; very-low-quality evidence) or linear/micronodular (focal) hyperplasia (OR 3.98; 95% CI 1.31 to 12.16; P value = 0.02; low-quality evidence).	Despite the uncertainty, there is a higher possibility of ECL cell hyperplasia during long-term maintenance prescription of PPIs.

Quality Assessment

The articles were assessed for eligibility using different quality appraisal tools. Table 3-7 shows the quality appraisal for the included case reports, narrative reviews, systematic reviews and meta-analyses, RCTs, and cohort studies, respectively.

**Table 3:** Quality appraisal of case reports using the JBI critical appraisal checklist for case reports Studies assessed using the JBI tool for case reports were labeled as ‘GOOD’ if they received a score of ≥5 out of 8 ‘YES’ responses, excluding ‘N/A’ items. This threshold aligns with a ≥60% quality score to be considered acceptable for inclusion. _Q1- Were patient’s demographic characteristics clearly described? Q2- Was the patient’s history clearly described and presented as a timeline? Q3- Was the current clinical condition of the patient on presentation clearly described? Q4- Were diagnostic tests or assessment methods and the results clearly described? Q5- Was the intervention(s) or treatment procedure(s) clearly described? Q6- Was the post-intervention clinical condition clearly described? Q7- Were adverse events (harms) or unanticipated events identified and described? Q8- Does the case report provide takeaway lessons? Q9- Overall quality_

Study, year	Q1	Q2	Q3	Q4	Q5	Q6	Q7	Q8	Q9
Hashimoto et al., 2014 [19]	NO	YES	YES	YES	YES	YES	YES	YES	GOOD
Imai et al., 2018 [20]	NO	YES	YES	YES	YES	YES	YES	YES	GOOD
Jianu et al., 2012 [21]	NO	YES	YES	YES	YES	YES	YES	YES	GOOD
Konijeti et al., 2013 [22]	NO	YES	YES	YES	YES	YES	YES	YES	GOOD
Kubo et al., 2020 [23]	NO	YES	YES	YES	YES	YES	YES	YES	GOOD
Takeda et al., 2017 [24]	NO	YES	YES	YES	YES	YES	N/A	YES	GOOD
Wu et al., 2014 [25]	NO	YES	YES	YES	YES	YES	YES	YES	GOOD
Miyamoto et al., 2017 [26]	NO	YES	YES	YES	YES	YES	YES	YES	GOOD
Nagao et al., 2024 [27]	NO	YES	YES	YES	YES	YES	YES	YES	GOOD
Nakashima et al., 2024 [28]	NO	YES	YES	YES	YES	YES	YES	YES	GOOD

**Table 4:** Quality assessment of included narrative reviews by using the SANRA checklist Based on the 12-point scale, quality was defined as follows: 10–12 = High quality, 8–9 = Moderate quality, <8 = Low quality. Only studies with a score of ≥8 were included. SANRA: Scale for the Quality Assessment of Narrative Review Articles _Q1) Justification of the article’s importance for the readership Q2) Statement of concrete aims or formulation of questions Q3) Description of the literature search Q4) Referencing Q5) Scientific reasoning Q6) Appropriate presentation of data _

Study, year	Q1	Q2	Q3	Q4	Q5	Q6	Sum Score	Interpretation of Quality
Lué and Lanas, 2016 [29]	2	2	1	2	2	2	11	High

**Table 5:** Quality assessment of included systematic reviews and meta-analysis using AMSTAR-2 AMSTAR: Assessment of Multiple Systematic Reviews

AMSTAR criteria	Boghossian et al., 2017 [30]	Pinto-Sanchez et al., 2017 [1]	Shanika et al., 2023 [5]	Song et al., 2014 [31]
Did the research questions and inclusion criteria for the review include the components of PICO?	YES	YES	YES	YES
Did the report of the review contain an explicit statement that the review methods were established prior to the conduct of the review and did the report justify any significant deviations from the protocol?	YES	YES	YES	YES
Did the review authors explain their selection of the study designs for inclusion in the review?	YES	YES	YES	YES
Did the review authors use a comprehensive literature search strategy?	YES	YES	YES	YES
Did the review authors perform study selection in duplicate?	YES	YES	YES	YES
Did the review authors perform data extraction in duplicate?	YES	YES	YES	YES
Did the review authors provide a list of excluded studies and justify the exclusions?	YES	YES	YES	YES
Did the review authors describe the studies included in adequate detail?	YES	YES	YES	YES
Did the review authors use a satisfactory technique for assessing the risk of bias in individual studies that were included in the review?	YES	YES	YES	YES
Did the review authors report on the sources of funding for the studies included in the review?	YES	YES	YES	YES
If a meta-analysis was performed, did the authors use appropriate methods to statistically combine results?	NOT APPLICABLE	NOT APPLICABLE	NOT APPLICABLE	NOT APPLICABLE
If a meta-analysis was performed, did the review authors assess the potential impact of risk of bias in individual studies on the results of the meta-analysis or other evidence synthesis?	NOT APPLICABLE	NOT APPLICABLE	NOT APPLICABLE	NOT APPLICABLE
Did the review authors account for the risk of bias in individual studies when interpreting/discussing the results of the review?	YES	YES	YES	YES
Did the review authors provide a satisfactory explanation for and discussion of any heterogeneity observed in the results of the review?	YES	YES	YES	YES
If they performed quantitative synthesis, did the review authors carry out an adequate investigation of publication bias (small study bias) and discuss its impact on the results of the review?	YES	YES	YES	YES
Did the review authors report any potential sources of conflict of interest, including any funding they received for conducting the review?	NO	NO	NO	NO
Total score (out of 16)	13/16	13/16	13/16	13/16
Overall quality	81.2%	81.2%	81.2%	81.2 %

**Table 6:** Bias assessment of included RCTs using ROB-2 Cochrane RoB2 Tool assesses five domains: domain 1 (bias arising from the randomization process), Domain 2 (bias due to deviations from intended interventions), domain 3 (bias due to missing outcome data), domain 4 (bias in the measurement of the outcome), and domain 5 (bias in the selection of the reported result(s)). Each domain is scored either a) low risk (✓), b) some concerns (±), or c) high risk (X). *Studies with "Overall high risk" (X) were not included in this review. RCT: randomized clinical trial

Study, year	Domain 1	Domain 2	Domain 3	Domain 4	Domain 5	Overall
Avşar et al., 2013 [57]*	X	X	X	✓	✓	X
Bajaj et al., 2014 [32]	✓	±	±	✓	✓	✓
Boyce et al., 2015 [33]	✓	✓	✓	✓	✓	✓
Jang et al., 2020 [10]	✓	X	X	✓	✓	✓
Xie et al., 2018 [2]	✓	X	X	✓	✓	✓

**Table 7:** Quality appraisal of the included cohort studies using the Newcastle-Ottawa Scale Each * represents a score. * = 1 point, ** = 2 points, and *** = 3 points. Study quality was assessed using the Newcastle-Ottawa Scale, where studies were rated on a scale of 0 to 9 stars. Studies scoring: 7–9 stars = High quality 6 stars = Moderate quality ≤5 stars = Low quality. Only moderate and high-quality studies were included. ^&^These studies were not included in the final review

Study, year	Selection	Comparison	Outcome	Score	Study Quality
Al Ali et al., 2022 [34]	****		**	6	Moderate
Al-Momani and Aolymat, 2024 [35]	****	*	**	7	High
Brito et al., 2018 [58]^&^	**		**	4	Low
Brunner et al., 2012 [36]	****		***	7	High
Contaldo et al., 2019 [37]	****	*	***	8	High
De Roza et al., 2019 [38]	****		***	7	High
Dilaghi et al., 2022 [39]	*****		***	8	High
Freedberg et al., 2013 [40]	*****	*	***	9	High
Fujimoto and Hongo, 2011 [3]	****		***	7	High
Hatano et al., 2016 [41]	*****		***	8	High
He et al., 2021 [4]	*****		**	7	High
Herzig et al., 2011 [42]	*****	*	***	9	High
Horvath et al., 2019 [43]	***	*	***	7	High
Iida et al., 2012 [44]	****		***	7	High
Jacobs et al., 2013 [9]	****		***	7	High
Kumarakulasinghe et al., 2016 [59]^$^	**		***	5	Low
Kurlander et al., 2020 [8]	*****		**	7	High
Kwon et al., 2014 [45]	****		***	7	High
Lassalle et al., 2022 [46]	***	*	***	7	High
Mafi et al., 2019 [6]	***		***	6	Moderate
Majima et al., 2018 [47]	*****		***	8	High
Nasser et al., 2015 [11]	****		***	7	High
Paroni Sterbini et al., 2016 [48]	****	*	***	8	High
Parsons et al., 2017 [49]	*****		***	8	High
Rodrigues et al., 2024 [50]	*****		***	8	High
Snir et al., 2021 [12]	*****		***	8	High
Takahari et al., 2017 [51]	****		***	7	High
Tatsuguchi et al., 2020 [52]	****		***	7	High
Tranberg et al., 2021 [53]	****		***	7	High
Valkhoff et al., 2012 [54]	*****		***	8	High
Wauters et al., 2021 [7]	****		***	7	High
Xie et al., 2017 [55]	*****		***	8	High
Yamada et al., 2017 [56]	****		***	7	High

Discussion

Microbiota Alterations in the GI System

One possible contributor to pathological changes in the GI system caused by PPIs is the alteration of gut bacteria. A clinical trial conducted on cirrhotic patients who consumed omeprazole revealed an increased incidence of *Streptococcaceae* bacteria using stool microbiota profiling with multi-tagged pyrosequencing (p=0.005). The microbiota composition predominantly changed in both the controls and cirrhotic patients (QIIME P <0.0001) [32]. Another gut bacterium that was found predominantly was *Veillonella *[43]. Both *Streptococcaceae* and *Veillonella* were linked to increased proinflammatory cytokines such as interleukin-6 (IL-6), chemokine ligand-8 (CXCL8), and tumor necrosis factor-alpha (TNF-α), leading to increased patient mortality risks [43]. De Roza et al. conducted a study in a Singapore hospital using data from January 2013 to June 2017 to assess mortality amongst PPI users, and reported an adjusted hazard ratio (HR) of 2.10 (95%CI: 1.20-3.67; P = 0.009), thus proving that the patients had a higher incidence of hospitalization [38]. More extended PPI usage, especially with cDDD> 90, increased the mortality significantly.

Gastric acid suppression leads to decreased pathogenic microbe elimination. One such microbe is *C. difficile*. In the presence of PPIs, they cause devastating pathological outcomes such as spontaneous gas gangrene and necrotizing fasciitis, leading to septicemia and death [25]. PPIs increase colonization of several multidrug-resistant bacteria and viremia and show 69% higher odds of recurrent *C. difficile* infection compared to those not taking PPIs [40]. A study conducted by Jacobs et al. showed a 40% incidence of small intestinal bacterial overgrowth (SIBO), 26% incidence of small intestinal fungal growth (SIFO), and 34% mixed incidence of these two categories in patients who used PPIs and had unexplained GI symptoms and negative endoscopy reports [9]. PPI is also found to have an association with disturbance in oropharyngeal microbiota. In a study by Tranberg et al., gut pathogens were colonized with an OR of 3.49, and the counts were directly proportional to the length of the hospital stay [53].

Although altered by PPIs, a cohort study by Parsons et al. showed that the microbial profile in the gut was not as severe as autoimmune atrophic gastritis and *H. pylori *atrophic gastritis, and the treated gut had a decrease in the level of *Acinetobacter* and *Tannerella* at the genus level [49]. An abundance of *Actinomycetales* gut bacteria increases the levels of celiac disease-specific autoantibody markers, and this was discovered through molecular mechanisms studying the association of PPI use and celiac disease through enhancement of gluten immunopathology [10]. These changes in the gut microbial flora have become an essential indicator in identifying patients who are overprescribed without necessity and therefore help regulate careful prescription of PPIs [48].

Prescription Trends and the Inappropriate Use of PPIs

Shanika et al. conducted a review on the global trends of PPI usage, which showed that one-quarter of adults globally use PPI, and out of those, 63% adults were less than age 65 years, where White ethnicities accounted for nearly 75% of users [5]. Polypharmacy has a higher chance of resulting in chronic PPI use without assessment of the risk-benefit aspect and has led to various drug interactions and infections. One challenging factor is the lack of patient satisfaction with the on-demand prescription of PPIs, which was determined by their unwillingness to continue PPIs [30]. Currently, it is estimated that 50% of PPI prescriptions are given for non-approved indications. For instance, patients taking NSAIDs for short-term pain management do not need gastroprotection to prevent lower GI bleeding [29]. High-risk patients, such as those with complicated ulcers or multiple risk factors, may need PPI to prevent GI bleeding, as the risk is increased by 9% (OR 1.09; 95%CI: 1.00-1.18) [54]. Specific populations, like older patients, need a careful risk-benefit assessment of PPI use. A large number of low-value prescriptions are issued to the elderly population, as seen in a study conducted from 2013 to 2019 on adults older than 65 years [6]. Initially, most of them were in the appropriately prescribed category (82%), which was for a duration of less than eight weeks; however, later they were categorized into the low-value category to 35% (95%CI = 31.3%-40.7%) as the patients had continued PPIs without an indication. PPI prescriptions lasting more than eight weeks are generally not recommended. A cross-sectional study conducted by Rodrigues et al. on 1200 older adults over 65 years of age demonstrated that 78.68% of them were taking PPIs for more than eight weeks, and 49.79% were taking them despite no GI indications [50]. Kurlander et al. conducted a multicenter study on 799 internists to assess the risk-benefit of PPI prescription, in which the majority of responders stated that PPIs indeed were causing multiple adverse events, and 86% recommended PPI discontinuation when not required [8]. PPIs are also not recommended for prophylactic use in non-critically ill hospitalized patients [42].

The Ill Effects of Long-Term PPI Use

A study by Brunner et al. gives an overview of PPIs' long-term safety and efficacy before discussing their adverse effects [36]. In their study, 142 adult patients received a maintenance dose of PPI (40-160 mg/day) for 15 years, and the study showed that the mean gastrin levels were increased, and the mean enterochromaffin cell counts rose initially for up to three years and remained stable thereafter. No changes in the gastric mucosa were identified using endoscopy, and long-term regression of antral and corpus gastritis was noted in the *H. Pylori* eradicated patient group. He et al., in their study, showed that PPI use does not increase all-cause mortality [4]. However, numerous adverse events have been linked with long-term PPI use. Consumption of PPIs during COVID-19 infection has resulted in GI adverse effects such as abdominal pain and diarrhea [35]. PPIs have been linked to increasing risk factors for pancreatic cancer [46]. Omeprazole has been reported to alter the hematological profile, especially red blood cells, resulting in anemia [34]. Case reports have shown that rabeprazole is associated with iron deficiency anemia in the adult population [19]. Imai et al. reported a case of a 52-year-old adult who was taking omeprazole for 25 years, who was diagnosed with iron deficiency anemia, and tests to rule out causes of IDA, such as upper GI endoscopy, fecal occult blood test, small intestine capsule endoscopy, and colonoscopy, were found to be negative [20]. This confirmed PPI as the reason due to poor absorption of iron as a result of gastric acid suppression by omeprazole.

Video capsule endoscopy is a great tool to detect unexplained IDA [37]. Another interesting adverse effect of PPI is that it is linked with colitis, and withdrawal of PPI heals the colitis in those affected [22]. The most important adverse events that need to be well-studied are the formation of polyps and malignancy. Long-term PPI use has been found to be strongly associated with multiple neuroendocrine tumors and gastric neoplastic lesions [27,39]. Jianu et al. reported the cases of two patients who were taking PPI for more than 10 years due to GERD and in whom an upper GI (UGI) scope revealed a solitary tumor in the oxyntic mucosa, where hyperplastic ECL cells were found [21]. Serum gastrin and chromogranin A are elevated during PPI use, and ECL levels decrease after discontinuing PPI, demonstrating PPI's risk factor for polyps and tumors [33]. ECL types linked with PPI are diffuse or linear/micronodular ECL hyperplasia [31]. A retrospective study published by Snir et al. showed upper quartiles of PPI doses had caused gastric intestinal metaplasia with a 5-10-fold increase in low-grade dysplasia [12].

Another noteworthy adverse event was the formation of gastric polyps and bleeding. A patient experienced uncontrollable bleeding from the polyp, which was characterized by capillary hyperplasia; the bleeding persisted despite multiple transfusions, and later, the cessation of PPI resulted in the polyp regressing [28]. In another case, where the patient presented with anemia and tarry stools, EGD showed multiple white, edematous polyps in the corpus and antrum [24]. The polyps showed foveolar epithelium hyperplasia, and proliferated parietal and chief cells were observed. Aquaporin-4 (AQP4) and KCNQ1-positive parietal cells and dilated mucous glands were found immunohistochemically.

However, certain studies have shown that PPI use does not lead to neuroendocrine tumors or gastric epithelial neoplasia despite ECL hyperplasia and small bowel injury [52,56]. A retrospective study conducted by Yamada et al. on 327 propensity-matched patient pairs suspected to have small bowel disease demonstrated no significant differences in the incidence of ulcers between users of PPI versus those who did not use it [56]. In general, overall results have proved that prolonged PPI use has been associated with an excessive risk of death even if the patients did not have any gastric condition [55]. Some other miscellaneous adverse events noted were that PPIs inhibit gastric perception in adults [44]. The adverse effect on the GI system caused by acid suppressants is not only limited to PPI but also extends to other groups of acid suppressants, such as potassium-competitive acid blockers. A case report published by Kubo et al. demonstrates that Vonoprazan caused gastric redness, visualized by an EGD scope [23]. Also, PPIs are associated with peritonitis in patients who are undergoing peritoneal dialysis [48]. PPIs cause multiple flat and white elevated lesions in the stomach and a cobblestone appearance in the mucosa, along with gastric lesions with black spots [26,41,47,50].

Limitations

It is important to disclose that our review has some restrictions. First, observational studies and a few number of RCTs make up the majority of the evidence related to the study. This could introduce prejudice and weaken the conclusions reached. Consistent recommendations are also challenging to develop due to differences in study design, doses, and the length of use of PPIs. A quantitative meta-analysis was not feasible due to the heterogeneity of included studies in terms of design, sample characteristics, and outcome reporting. This limits the ability to statistically pool effect sizes but allows for a broader narrative understanding of long-term PPI-related GI outcomes.

## Conclusions

This systematic review explored the harmful effects on the GI system after using PPIs for a long duration. Based on the studies reviewed, PPIs demonstrate an increased risk of numerous adverse events, such as infections, polyps, malignancy, and iron deficiency caused by poor absorption due to decreased gastric secretions. Additional well-studied randomized controlled studies are required to confirm the results and show a substantial causal link between long-term PPI usage and adverse effects on the GI tract. While PPI remains the most effective first-line drug for treating acid peptic disease, physicians need to carefully assess the risk-benefit factors and discontinue the drug when not required. Longitudinal studies are essential to assess the long-term adverse events of PPIs on the GI system in identifying the optimal duration for gastric acid suppression and reducing the risk associated with their use.
